# Demographics and survival of AIDS cases with cancer, Washington, DC, 1996-2006

**DOI:** 10.1186/1750-9378-7-S1-P5

**Published:** 2012-04-19

**Authors:** Heather A Young, Eric Engels, Ann-Marie Sufian-Kargbo, Alicia Vargas, Kathleen Rogers, Tiffany West, Amanda Castel

**Affiliations:** 1 Department of Epidemiology and Biostatistics, The George Washington University School of Public Health and Health Services, Washington, DC, USA; 2 DC Cancer Registry, Community Health Administration, District of Columbia Department of Health, Washington, DC, USA; 3 HIV/AIDS, Hepatitis, STD, TB Administration, District of Columbia Department of Health, Washington, DC, USA; 4 Infections and Immunoepidemiology Branch, Division of Cancer Epidemiology and Genetics, National Cancer Institute, National Institutes of Health, Rockville, MD, USA

## Background

Washington, DC (DC) has one of the highest HIV/AIDS rates in the U.S and cancer is the second leading cause of death among DC residents. This study sought to examine the demographic characteristics and survival of persons with AIDS defining cancers (ADCs) compared to those with non-AIDS defining cancers (NADCs) between the early HAART era (1996-2001) and the late HAART era (2002-2006) in DC.

## Methods

Cases reported from 1996-2006 to the DC Cancer Registry and the AIDS Surveillance Registry were linked using a probabilistic matching algorithm. Cases were included if the cancer occurred from 4 months to 60 months post-AIDS diagnosis and were stratified into ADCs and NADCs for analyses. Cancer diagnoses were stratified into the early and late HAART eras to compare the availability of HAART on the distribution of cancer type. Kaplan-Meier survival analysis and adjusted Cox proportional hazards regression were used to assess survival time and risk of death by cancer type.

## Results

From 1996-2006, among 8,800 AIDS cases, 300 (3.4%) cases had a cancer diagnosis. NADCs accounted for 51% of cancers and were significantly more likely to be diagnosed with AIDS (p<0.0001) and cancer (p<0.0001) at 40 years or older and had a significantly longer median time from AIDS to cancer diagnosis (2.46 vs. 1.75 years, p=0.01) compared to ADCs. The most common ADCs were Kaposi sarcoma (40%) and non-Hodgkin lymphoma (NHL) (44%); the most common NADC cases were lung (20%), Hodgkin lymphoma (8%) and anal (8%) cancer. ADCs accounted for 56% of cancer cases in the late-HAART as compared to the early-HAART period (45%). Mortality within the first year of cancer diagnosis was similar (ADC 41% vs. NADC 37%) and no statistical difference in survival time was observed. In the adjusted model, NHL and lung cases were significantly more likely to die as compared to other cancers (NHL HR=3.06; Lung HR=3.44).

## Conclusions

In DC, despite high HIV/AIDS and cancer prevalence, only a small proportion of AIDS cases also develop cancer with ADCs and NADCs being equally common. HAART availability does not seem to have altered survival among ADCs and NADCs. Survival among NHL cases was relatively low reflecting the need for increased access to care among HIV+ persons. NADC cases are most likely developing cancers related to advancing age with higher proportions of lung cancers being observed. Public health efforts should focus on lung cancer prevention and continued monitoring of HIV-infected persons for cancers.

